# Cardiac‐specific overexpression of metallothionein attenuates L‐NAME‐induced myocardial contractile anomalies and apoptosis

**DOI:** 10.1111/jcmm.14375

**Published:** 2019-05-18

**Authors:** Lifang Yang, Jipeng Ma, Ying Tan, Qijun Zheng, Maolong Dong, Wei Guo, Lize Xiong, Jian Yang, Jun Ren

**Affiliations:** ^1^ Department of Anesthesiology Xi'an Children Hospital Xi'an China; ^2^ Center for Cardiovascular Research and Alternative Medicine University of Wyoming Laramie Wyoming; ^3^ Department of Cardiovascular Surgery, Xijing Hospital Fourth Military Medical University Xi'an China; ^4^ Department of Emergency Medicine, Nanfang Hospital Southern Medical University Guangzhou China; ^5^ Department of Burns, Nanfang Hospital Southern Medical University Guangzhou China; ^6^ Department of Animal Sciences University of Wyoming Laramie Wyoming; ^7^ Department of Anesthesiology, Xijing Hospital Fourth Military Medical University Xi'an China

**Keywords:** apoptosis, heart, hypertension, L‐NAME, metallothionein

## Abstract

Hypertension contributes to the high cardiac morbidity and mortality. Although oxidative stress plays an essential role in hypertensive heart diseases, the mechanism remains elusive. Transgenic mice with cardiac overexpression of metallothionein, a heavy metal‐binding scavenger, were challenged with N^G^‐nitro‐L‐arginine methyl ester (L‐NAME) for 14 days prior to measurement of myocardial contractile and intracellular Ca^2+^ anomalies as well as cell signalling mechanisms using Western blot and immunofluorescence analysis. L‐NAME challenge elicited hypertension, macrophage infiltration, oxidative stress, inflammation and cardiac dysfunction manifested as increased proinflammatory macrophage marker F4/80, interleukin‐1β (IL‐1β), intracellular ${\text{O}}_{2}^{-}$ production, LV end systolic and diastolic diameters as well as depressed fractional shortening. L‐NAME treatment reduced mitochondrial membrane potential (MMP), impaired cardiomyocyte contractile and intracellular Ca^2+^ properties as evidenced by suppressed peak shortening, maximal velocity of shortening/relengthening, rise in intracellular Ca^2+^, along with elevated baseline and peak intracellular Ca^2+^. These unfavourable mechanical changes and decreased MMP (except blood pressure and macrophage infiltration) were alleviated by overexpression of metallothionein. Furthermore, the apoptosis markers including BAD, Bax, Caspase 9, Caspase 12 and cleaved Caspase 3 were up‐regulated while the anti‐apoptotic marker Bcl‐2 was decreased by L‐NAME treatment. Metallothionein transgene reversed L‐NAME‐induced changes in Bax, Bcl‐2, BAD phosphorylation, Caspase 9, Caspase 12 and cleaved Caspase 3. Our results suggest that metallothionein protects against L‐NAME‐induced myocardial contractile anomalies in part through inhibition of apoptosis.

## 1 INTRODUCTION

Hypertension is a devastating cause of cardiovascular diseases which compromises cardiac structure and function.^1^, ^2^ It is well perceived that hypertension contributes to the ever rising risk of cardiovascular morbidity and mortality through onset and development of heart disease in addition to the well‐established vascular anomalies.^2^, ^3^, ^4^ A plethora of clinical and experimental studies has depicted the unique role of apoptosis in hypertensive heart disease.^5^, ^6^, ^7^ Furthermore, reactive oxygen species (ROS) can directly regulate the apoptosis, mitochondrial integrity and survival in the hearts.^8^ Indeed, antioxidant treatment has been shown to retard oxidative stress, mitochondrial injury and apoptotic cell death in pulmonary arterial hypertension‐induced heart failure, suggesting the therapeutic value of antioxidants in treatment of cardiac diseases associated pulmonary hypertension.^9^, ^10^, ^11^ It was also suggested that the mitochondria‐targeted antioxidant peptide SS‐31 alleviated mitochondrial oxidative injury and geometric hypertrophy in angiotensin II‐induced hypertensive cardiomyopathy.^12^ Therefore, manipulation of ROS through antioxidants and mitochondria may display some promises in the management of cardiac dysfunction in hypertensive heart disease. To this end, this study was designed to examine the impact of metallothionein in hypertension‐induced changes of cardiac remodelling, inflammation, contractile function and cell apoptosis. Metallothioneins belong to group of low molecular weight intracellular cysteine‐rich heavy metal‐binding proteins.^13^ Up‐to‐date, a variety of biological actions have been reported for metallothioneins including serving as potent antioxidants and free radical scavengers against reactive oxygen and nitrogen species including superoxide anions, hydrogen peroxide and peroxynitrite in pathological conditions such as obesity, cigarette smoking and diabetes.^14^, ^15^, ^16^, ^17^ In addition, data from our group suggested that metallothionein also preserved cardiac function via autophagy inhibition in cardiac dysfunction resulted from N^G^‐nitro‐L‐arginine methyl ester hydrochloride (L‐NAME)‐induced hypertension.^18^ Hence, the NO synthase inhibitor L‐NAME was employed to generate experimental hypertensive model.^19^, ^20^ To discern the role of macrophage infiltration, inflammation, oxidative stress, apoptosis in metallothionein‐ and hypertension‐induced cardiac dysfunction, levels of macrophage infiltration marker F4/80, inflammation marker interleukin‐1β (IL‐1β), superoxide (${\text{O}}_{2}^{-}$) production and apoptotic markers Bax, bcl‐2, phosphorylated BAD, Caspase 9 and Caspase 12 and cleaved Caspase 3 were evaluated in wild‐type and metallothionein transgenic mice treated with L‐NAME. Hypertension is associated with profound macrophage infiltration and inflammation, leading to cell and organ damage.^21^, ^22^ Furthermore, levels of Tenascin C and osteopontin‐1 were monitored for any potential cardiac remodelling process. JC‐1 staining was employed to assess the mitochondrial membrane potential (MMP). TUNEL staining was used to assess apoptosis in hearts from FVB and metallothionein transgenic mice with or without L‐NAME treatment.

## 2 MATERIALS AND METHODS

### 2.1 Experimental animals and hypertension model

All experimental procedures described here were approved by the Animal Care and Use Committees of the University of Wyoming (Laramie, WY, USA) and the Fourth Military Medical University (Xi'an, China). Transgenic mice with ~10‐fold cardiac‐specific overexpression of metallothionein (type IIa) were described in details in previous studies.^23^, ^24^ Five‐to‐6‐month‐old male transgenic mice and wild‐type friend virus B (FVB) mice were provided 0.1 g/L N^G^‐Nitro‐L‐arginine Methyl Ester (L‐NAME) in drinking water for 14 days to induce hypertension.^19^ Treatment of L‐NAME for 14 days has been used routinely to induce experimental hypertension.^25^, ^26^ All mice were housed in a climate‐controlled environment at 22.8 ± 2.0°C, 45%‐50% humidity, under a 12/12‐light/dark cycle with free access to food.

### 2.2 Blood pressure and echocardiographic assessment

Conscious systolic and diastolic blood pressures were measured using a KODA semi‐automated non‐invasive blood pressure device (Kent Scientific Corp, Torrington, CT, USA).^27^ Cardiac geometry and function were evaluated in anaesthetized (80 mg/kg ketamine and 12 mg/kg xylazine, ip) mice using two‐dimensional guided M‐mode echocardiography (Sonos 5500) equipped with a 15‐6 MHz linear transducer. Left ventricular (LV) anterior and posterior wall dimensions during diastole (LVPWd) and systole (LVPWs) were recorded from three consecutive cycles in M‐mode using methods adopted by the American Society of Echocardiography. Fractional shortening was calculated from LV end diastolic (LVEDD) and end systolic (LVESD) diameters according to the equation (LVEDD‐LVESD)/LVEDD. Heart rates were averaged from 20 consecutive cycles.^28^

### 2.3 Isolation of murine cardiomyocytes

Cardiomyocytes were freshly isolated as described previously.^29^ After ketamine/xylazine sedation (ketamine 80 mg/kg and xylazine 12 mg/kg xylazine, ip), hearts were removed and perfused with Krebs‐Henseleit bicarbonate (KHB) buffer at room temperature containing (in mM): 118 NaCl, 4.7 KCl, 1.2 MgSO_4_, 1.2 KH_2_PO_4_, 25 NaHCO_3_, 10 HEPES and 11.1 glucose. Solution was gassed with 5% CO_2_/95% O_2_. Hearts were isolated using liberase enzymatic digestion (Hoffmann‐La Roche Inc, Indianapolis, IN, USA) for 20 minutes. Digested hearts were removed from the cannula and left ventricle was cut into small pieces. Undigested tissues were gently agitated and cell pellet was resuspended. Extracellular Ca^2+^ was added to a final concentration of 1.2 mmol/L gradually over a period of 30 minutes. Only rod‐shaped myocytes with clear sarcomere striations were selected for mechanical properties and intracellular Ca^2+^ study.^30^

### 2.4 Cell shortening/relengthening

Mechanical properties of isolated cardiomyocytes were evaluated using an IonOptix™ soft‐edge system (IonOptix, Milton, MA, USA). Cardiomyocytes were laid in a chamber mounted on the stage of an Olympus IX‐70 inverted microscope and superfused (~2 mL/min at 25°C) with a Krebs‐Henseleit bicarbonate buffer containing (in mmol/L) 131 NaCl, 4 KCl, 1 MgCl_2_, 1 CaCl2, 10 HEPES and 10 glucose at pH 7.4. Myocytes were stimulated at a frequency of 0.5 Hz using a pair of platinum wires on the opposite sides of the chamber connected to a FHC stimulator (Brunswick, NE, USA). The cell was shown on the computer monitor by an IonOptix MyoCam camera. Cell shortening and relengthening were assessed by the following indices: resting cell length, peak shortening (PS), time‐to‐PS (TPS), time‐to‐90% relengthening (TR_90_) and maximal velocities of shortening/relengthening (±dL/dt).^28^

### 2.5 Intracellular Ca^2+^ transients

Fura‐2/AM (0.5 µmol/L) was added to a cohort of isolated cardiomyocytes for 10 minutes incubation and fluorescence intensity was measured with a dual‐excitation fluorescence photomultiplier system (IonOptix). Cardiomyocyte was placed onto an Olympus IX‐70 inverted microscope and imaged through a Fluor 40x oil objective. Cardiomyocytes were exposed to light emitted by a 75W lamp and passed through either a 360 or a 380 nm filter, while being stimulated to contract at a frequency of 0.5 Hz. Fluorescence emissions were detected between 480 and 520 nm and qualitative change in fura‐2 fluorescence intensity (FFI) was deduced from the FFI ratio at the two wavelengths (360/380). Fluorescence decay time (single exponential) was calculated as an indicator of intracellular Ca^2+^ clearing.^31^

### 2.6 Intracellular fluorescence measurement of ${\text{O}}_{2}^{-}$

Intracellular ${\text{O}}_{2}^{-}$ was monitored by changes in fluorescence intensity from intracellular probe oxidation. In brief, freshly isolated cardiomyocytes were loaded with 5 μmol/L dihydroethidium (DHE) (Molecular Probes, Eugene, OR) for 30 minutes at 37°C. Cells were evaluated using an Olympus BX‐51 microscope with Olympus MagnaFire™ SP digital camera. Fluorescence was calibrated using InSpeck microspheres (Molecular Probes) and was quantitated using an ImagePro analysis software (Media Cybernetics, Silver Spring, MD).^32^

### 2.7 TUNEL staining

Frozen myocardial sections were obtained from the mid‐section of the whole heart (about half way between apex and mitral valves) and were stained with a TUNEL staining kit (Roche). Sections were rinsed in PBS buffer for 10 minutes before incubation with proteinase K (20 μg/mL) in Tris‐HCl for 30 minutes at 37°C. In a humidified chamber, sections were incubated with the anti‐Desmin antibody (Cell Signaling, 1:50) at 4°C overnight. After that, sections were incubated with anti‐Rabbit secondary conjugate with Alex Fluor 568 (Invitrogen) at 37°C for 1 hour. Sections were then incubated with the TUNEL staining solution at 37°C for 1 hour in a humidified chamber. Following PBS wash, myocardial sections were stained with DAPI for 10 minutes at 37°C in a humidified chamber. Sections were mounted with 50% glycerol and were used for fluorescent detection using confocal microscopy. The percentage of apoptotic cells in total cells was calculated.^33^, ^34^

### 2.8 Immunofluorescent staining of myocardial tissues

The anti‐F4/80 antibody was used to label macrophages to examine the inflammatory response triggered by L‐NAME. In brief, frozen myocardial sections (mid‐section from the whole heart) were gently mixed in PBS buffer for 10 minutes and then incubated in proteinase K (20 μg/mL) solution at 37°C for 30 minutes. Goat serum was added for blocking at 37°C for 1 hour. After rinsing in PBS, samples were incubated with anti‐F4/80 (1:100) antibody at 4°C overnight. To remove the excess primary antibody, sections were washed in PBS three times for 10 minutes each. Samples were then incubated with Alex Fluor 568 secondary antibody for 2 hour at room temperature. Cellular nuclei were stained with DAPI (10 μg/mL) for 15 minutes at room temperature. Slides were finally imaged using a confocal microscopy and the F4/8‐positive cells were expressed as a percentage of total cell numbers.^35^

### 2.9 Measurement of mitochondrial membrane potential (ΔΨm)

Given that mitochondrial function is key to cardiomyocyte viability, the cationic lipophilic probe JC‐1 was employed to measure ΔΨm. The dynamic change of ΔΨm was displayed by change in the ratio between red (aggregated JC‐1) and green (monomeric JC‐1) fluorescence. Murine cardiomyocytes (with a viability of 75%‐80%) were suspended in HEPES‐saline buffer and mitochondrial membrane potential (ΔΨm) was detected as described. Briefly, following a 10‐min preincubation with 5 μmol/L JC‐1 at 37°C, cells were rinsed twice by sedimentation using HEPES buffer free of JC‐1. The cardiomyocytes were observed by a confocal microscopy. Fluorescence was read at excitation wavelength of 490 nm and emission wavelength of 530 and 590 nm using a spectrofluorimeter (SpectraMaxGeminiXS, Spectra Max, Atlanta, GA, USA). Results in fluorescence intensity were expressed as 590 to 530 nm emission ratio. The uncoupler carbonyl cyanide m‐chlorophenylhydrazone (CCCP, 10 μmol/L) was employed as a positive control for mitochondrial membrane potential.^36^, ^37^

### 2.10 Caspase‐3 activity assay

Caspase 3 activity was measured according to the previously published method.^31^, ^33^ In Brief, 1 mL of PBS was added to whole heart tissues. Then the tissues were homogenized and centrifuged at 10 000 × *g* for 10 minutes at 4°C. The supernatant was discarded and pellets were lysed in 100 μL of ice‐cold lysis buffer (50 mmol/L HEPES, pH 7.4, 0.1% CHAPS, 1 mmol/L dithiothreitol (DTT), 0.1 mmol/L EDTA, 0.1% NP40). The assay for Caspase 3 activity was carried out in a 96‐well plate. Each well contained 30 μL of lysate, 70 μL of assay buffer (50 mmol/L HEPES,pH 7.4, 0.1% CHAPS, 100 mmol/L NaCl, 10 mmol/L DTT and 1 mmol/L EDTA) and additional 20 μL of Caspase 3 colorimetric substrate Ac‐DEVD‐pNA. The 96‐well plate was incubated at 37 ^◦^C for 2 hours, during the time Caspase in the sample was allowed to cleave the chromospheres pNA from the substrate molecule. Absorbance readings were obtained at 405 nm with the Caspase 3 activity being directly proportional to the colorimetric reaction. Protein concentration was determined using the Bradford method. The final Caspase 3 activity was expressed as picomoles of pNA released per microgram of protein per minute.^33^, ^38^

### 2.11 Western blot analysis

Whole heart protein samples containing equal amount of proteins were loaded on 10% SDS‐polyacrylamide gels in a minigel apparatus (Mini‐PROTEAN II, Bio‐Rad, Hercules, CA, USA) and transferred to nitrocellulose membranes (0.2 μm pore size, Bio‐Rad). The membranes were blocked with 5% milk in TBS‐T for 2 hours and were incubated overnight at 4°C with anti‐metallothionein, anti‐BAX, anti‐phospho‐BAD, anti‐BAD, anti‐Bcl2, anti‐Caspase 9, anti‐Caspase 12, anti‐cleaved Caspase 3, anti‐tenascin C, anti‐osteopontin‐1, anti‐IL‐1β, anti‐ copper/zinc superoxide dismutase‐1 (SOD1), anti‐heme oxygenase (HMOX‐1) (SOD1 and HMOX‐1 were used to assess antioxidant capacity) and anti‐GAPDH (loading control) antibodies. After incubation and washing steps, the secondary antibodies conjugated with horseradish peroxidase (HRP) were added. The proteins were detected by enhanced chemiluminescence (Amersham Pharmacia, Piscataway, NJ, USA) and quantified by Quantity One software (Bio‐Rad, version 4.6.2). GAPDH was used as the loading control.

### 2.12 Statistical analysis

All statistical analysis was performed with GraphPad Prism 4.0 software. Data were expressed as Mean ± SEM. Statistical significance (*P* < 0.05) was estimated by one‐way analysis of variance (ANOVA) followed by a Turkey's test for *post hoc* analysis.

## 3 RESULTS

### 3.1 General biometric features, blood pressure and echocardiographic properties

**Figure 1:**
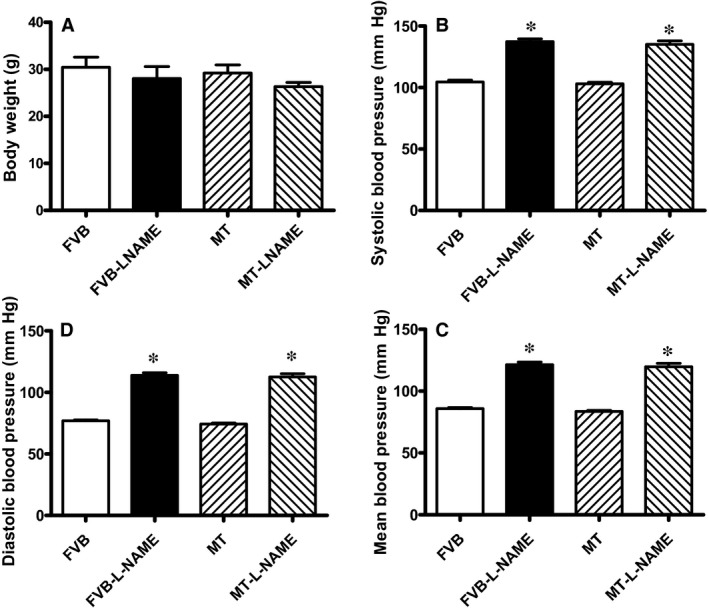
Blood pressure in FVB and metallothionein (MT) mice with or without L‐NAME: A: Body weight; B: Systolic blood pressure; C: Diastolic blood pressure; and D: Mean blood pressure. Mean ± SEM, n = 10‐12 mice per group, *P < 0.05 vs FVB group

**Figure 2:**
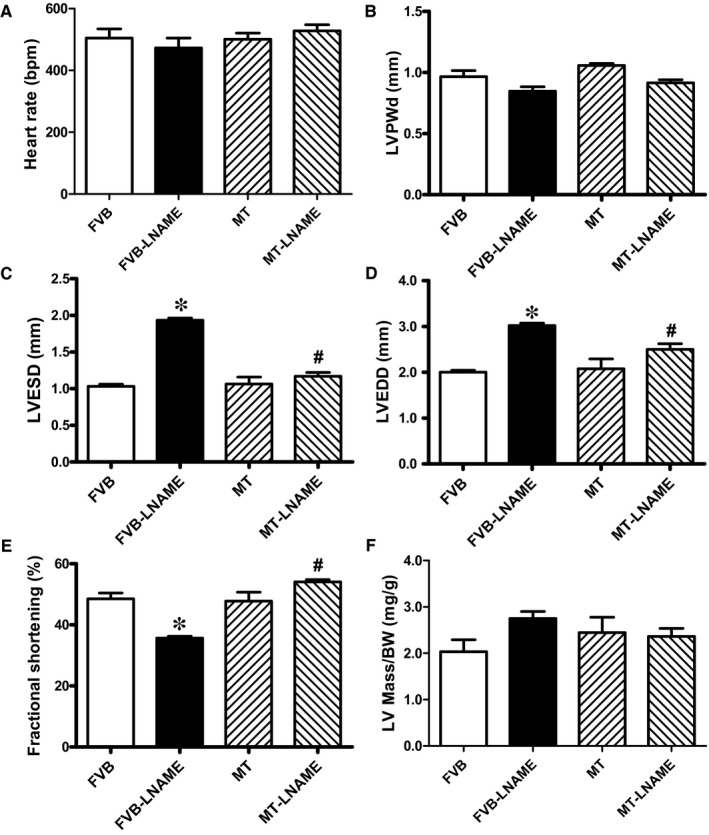
Effect of metallothionein (MT) on L‐NAME‐induced echocardiographic changes: A: Heart rate; B: LV posterior wall thickness during diastole (LVPWd): C: LV end systolic diameter (LVESD): D: LV end diastolic diameter (LVEDD); E: Fractional shortening; and F: LV mass (normalized to body weight). Mean ± SEM, n = 8‐10 mice per group, *P < 0.05 vs FVB group, #*P* < 0.05 vs FVB‐L‐NAME group

Our data shown in Figure 1 revealed that treatment of 0.1 g/L L‐NAME in drinking water for 14 days significantly increased diastolic, systolic and mean blood pressures in both FVB and MT mice, in a comparable manner. Cardiac overexpression of metallothionein alone did not affect blood pressures. Neither L‐NAME treatment nor metallothionein transgene affected body weight. As demonstrated in Figure 2, echocardiographic assessment suggested that L‐NAME treatment dramatically increased LV end systolic diameter (LVESD) and LV end diastolic diameter (LVEDD) accompanied with diminished fractional shortening, the effect of which was negated by metallothionein transgene. Metallothionein transgene itself did not display any detectable effect on these echocardiographic parameters. Neither L‐NAME treatment nor metallothionein, or both, affected heart rate, LV wall thickness and LV mass (normalized to body weight).

### 3.2 Effect of metallothionein on L‐NAME‐induced changes in cardiomyocyte contractile and intracellular Ca^2+^ properties as well as intracellular ${\text{O}}_{2}^{-}$ production

**Figure 3:**
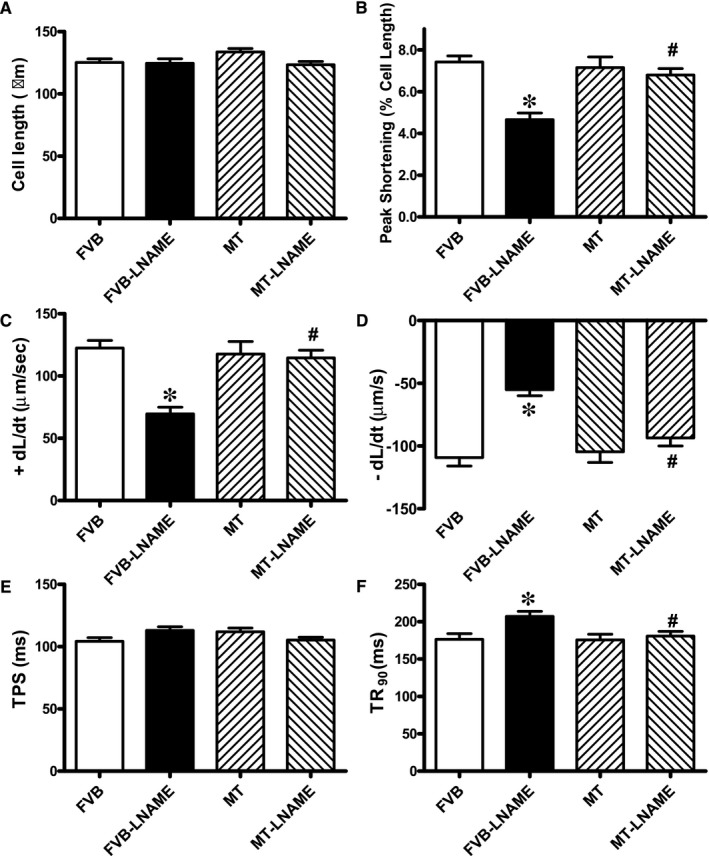
Effect of L‐NAME on cardiomyocyte contractile properties in FVB and MT mice: A: Resting cell length; B: Peak cell shortening (PS, normalized to resting cell length); C: Maximal velocity of cell shortening (+dL/dt); D: Maximal velocity of relengthening (−dL/dt); E: Time‐to‐peak shortening (TPS); and F: Time‐to‐90% relengthening (TR_90_). Mean ± SEM, n = 78‐85 cells per group, *P < 0.05 vs FVB group, #*P* < 0.05 vs FVB‐L‐NAME group

**Figure 4:**
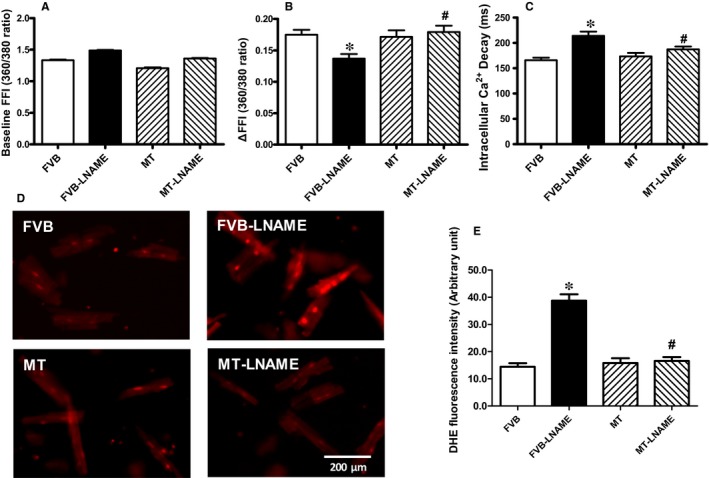
Effect of L‐NAME on cardiomyocyte intracellular Ca^2+^ and ${\text{O}}_{2}^{-}$ properties in FVB and MT mice: A: Baseline fura‐2 fluorescence intensity (FFI); B: Rise in intracellular Ca^2+^ in response to electrical stimulus (ΔFFI); C: Intracellular Ca^2+^ decay rate; D: Representative intracellular fluorescent images depicting ${\text{O}}_{2}^{-}$ levels using DHE staining in four experimental groups; and E: Pooled data for ${\text{O}}_{2}^{-}$ production. Mean SEM, n = 52‐65 cells per group (panel A‐C) or n = 10 images per group for panel E, *P < 0.05 vs FVB group, #*P* < 0.05 vs FVB‐L‐NAME group

Neither L‐NAME treatment nor metallothionein transgene exhibited any overt effect on resting cardiomyocyte cell length. Fourteen days of L‐NAME treatment significantly decreased peak shortening and maximal velocity of shortening/relengthening (±dL/dt) as well as prolonged time‐to‐90% relengthening (TR_90_) without affecting time‐to‐PS (TPS). Although metallothionein itself failed to affect these cardiomyocyte mechanical indices, it abrogated L‐NAME‐induced mechanical alterations (Figure 3). To further elucidate the possible mechanisms underlying L‐NAME‐ and metallothionein‐induced cardiac mechanical changes, intracellular Ca^2+^ and intracellular ${\text{O}}_{2}^{-}$ were measured using the intracellular fluorescent dyes Fura‐2 and DHE respectively. Data presented in Figure 4A‐C displayed that L‐NAME treatment significantly suppressed electrically stimulated rise in fura‐2 fluorescence intensity (ΔFFI) and increased intracellular Ca^2+^ decay (longer decay time) with little effect on baseline FFI levels. Although metallothionein itself did not affect these intracellular Ca^2+^ parameters, it rescued against L‐NAME‐induced changes in ΔFFI and intracellular Ca^2+^ decay rate. In addition, L‐NAME significantly promoted intracellular ${\text{O}}_{2}^{-}$ production, the effect of which was obliterated by metallothionein (with little effect from metallothionein itself (Figure 4D‐E).

### 3.3 Effect of metallothionein on L‐NAME‐induced mitochondrial damage

**Figure 5:**
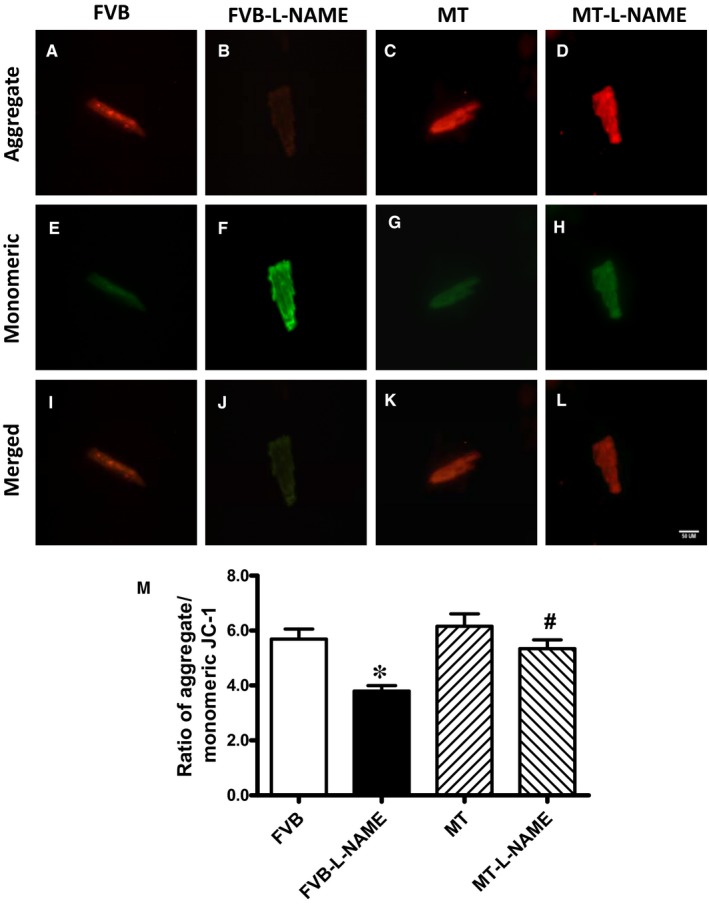
Effect of L‐NAME challenge on mitochondrial membrane potential measured using JC‐1 fluorescence: A‐D: Red fluorescence indicates hyperpolarized (J‐aggregate) mitochondria; E‐H: Green fluorescence indicates depolarized (monomer) mitochondria; I‐L: Merged red fluorescence and green fluorescence; and M. Quantification of ΔΨm expressed as ratio of aggregate/monomer fluorescence. Mean ± SEM, n = 20 cells per group, *P < 0.05 vs FVB group, #*P* < 0.05 vs FVB‐L‐NAME group

To explore the potential role of mitochondrial integrity in L‐NAME‐induced cardiac dysfunction, the cationic lipophilic probe JC‐1 was employed to monitor mitochondrial membrane potential (ΔΨm) in cardiomyocytes from FVB and metallothionein mice with or without L‐NAME treatment. Alteration of ΔΨm was reflected by changes of ratio between red (aggregated JC‐1) and green (monomeric form of JC‐1) fluorescence. Results shown in Figure 5 depicted an overt reduction in the ratio between red and green fluorescence in response to L‐NAME challenge, illustrating a drop in ΔΨm which represents mitochondrial injury. Intriguingly, the L‐NAME‐induced reduction of mitochondrial ΔΨm was ablated by the heavy metal scavenger while metallothionein alone fails to exhibit any effect on ΔΨm. These data demonstrated that the heavy metal scavenger alleviated L‐NAME‐induced mitochondrial injury.

### 3.4 Effect of metallothionein on L‐NAME‐induced apoptosis

**Figure 6:**
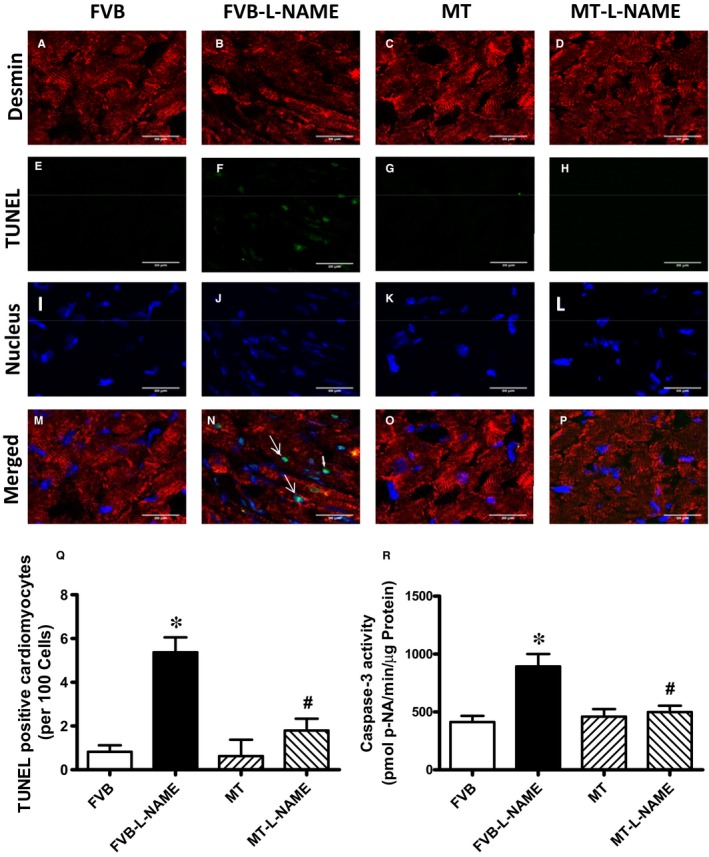
Confocal microscopic images depicting L‐NAME‐induced myocardial apoptosis: A‐P: Myocardial sections from WT and MT mice with or without L‐NAME treatment were stained with desmin (red), TUNEL (green) and nucleus with DAPI (blue). A‐D: Red fluorescence indicates cardiomyocytes; E‐H: Green fluorescence indicates apoptosis; I‐L: Blue fluorescence indicates nucleus; M‐P: are the merged images of the respective lines; Q, Quantitative analysis of apoptosis using TUNEL staining; and R: Caspase 3 activity. Mean ± SEM, n = 30 fields from 3 mice per group, *P < 0.05 vs FVB group, #*P* < 0.05 vs FVB‐L‐NAME group. DAPI = 4', 6‐diamidino‐2‐phenylindole; TUNEL = terminal dUTP nick end labelling

**Figure 7:**
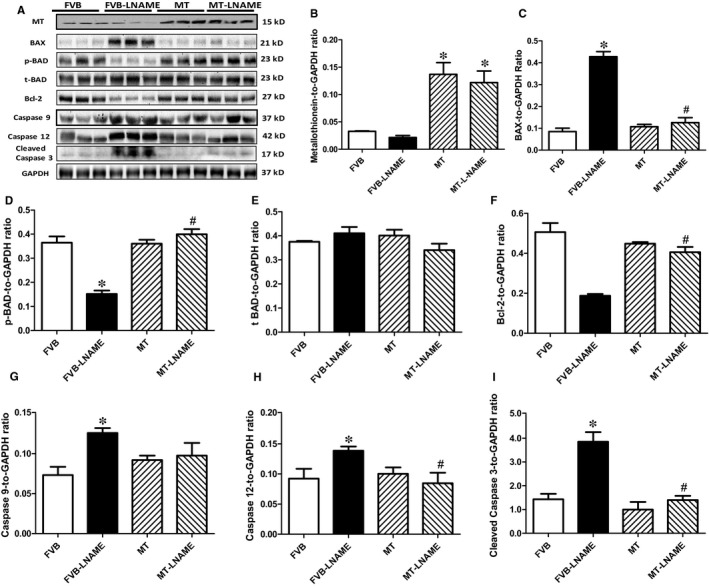
Western blot analysis of metallothionein (MT) and apoptotic markers in myocardium from FVB and MT mice treated with or without L‐NAME: A: Representative gel blots depicting expression of MT, BAD, phosphorylated BAD, Bcl‐2, Bax, Caspase 9, Caspase 12, cleaved Caspase 3 and GAPDH (used as the loading control); B: MT; C: Bax; D: p‐BAD‐to‐BAD ratio; E: BAD; F: Bcl‐2; G: Caspase 9; H: Caspase 12; and I: Cleaved Caspase 3. Mean ± SEM, n = 4‐5 mice per group, *P < 0.05 vs FVB group, #*P* < 0.05 vs FVB‐L‐NAME group

To further evaluate if apoptosis plays a role in metallothionein‐ and L‐NAME‐induced changes in myocardial function, apoptosis was examined in myocardium from FVB and metallothionein transgenic mice using TUNEL staining, Caspase 3 assay and Western blot. TUNEL staining revealed a greater percentage of TUNEL‐positive cells in L‐NAME‐induced FVB mice, the effect of which was ablated by metallothionein with little notable effect from the transgene itself. These findings were consolidated by Caspase 3 activity assay where metallothionein nullified L‐NAME‐induced rise in Caspase 3 activity (Figure 6). Our results further demonstrated that the level of cleaved Caspase 3 was increased in L‐NAME‐induced FVB mice while metallothionein overexpression significantly attenuated the up‐regulation of cleaved Caspase 3 (Figure 7H). Results of Western blot shown in Figure 7A‐B also revealed that L‐NAME treatment did not affect the levels of metallothionein in either FVB or MT mice. MT overexpression was verified using Western blot analysis. However, L‐NAME challenge overtly promoted levels of the pro‐apoptotic proteins BAX, Bad (reduced phosphorylation), Caspase 9 and Caspase 12 as well as down‐regulated anti‐apoptotic protein Bcl‐2. Consistent with the findings from TUNEL staining and Caspase 3 activity assays, metallothionein transgene protected against L‐NAME‐induced changes in these apoptotic protein markers without eliciting any effects by itself (Figure 7A‐H).

### 3.5 Effects of metallothionein on L‐NAME‐induced cardiac remodelling and inflammation

**Figure 8:**
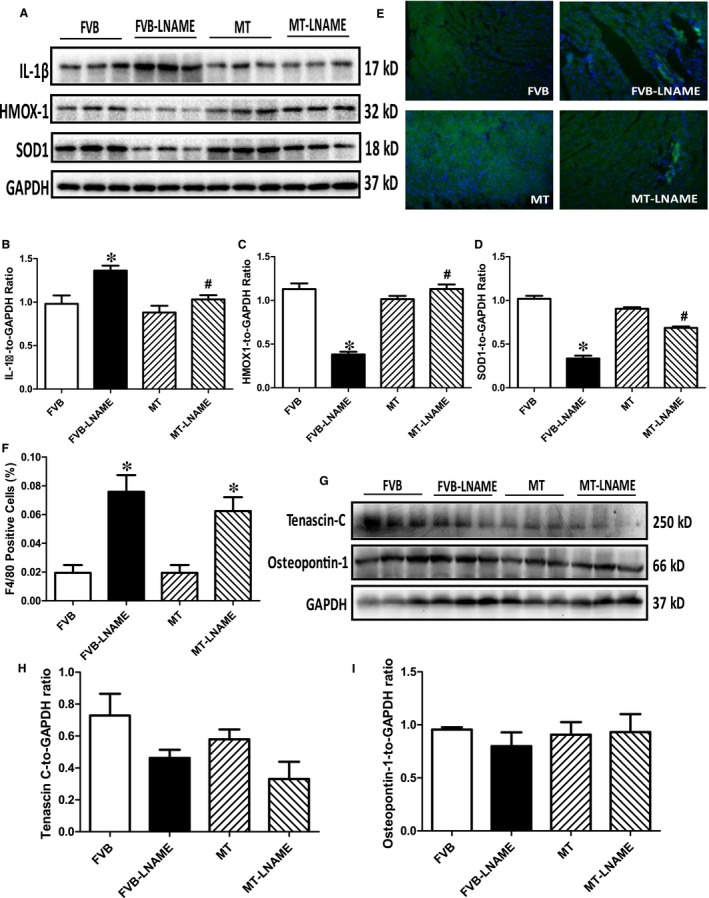
Western blot analysis of antioxidant and cardiac remodelling markers and immunofluorescent staining of proinflammatory macrophage in myocardium from FVB and metallothionein (MT) mice treated with or without L‐NAME: A: Representative gel blots depicting expression of IL‐1β, HMOX‐1, SOD1 and GAPDH (as the loading control); B: IL‐1β; C:HMOX‐1; D:SOD1; E: Representative images of immunofluorescent staining of myocardial macrophage with F4/80 antibody; F: Pooled data of F4/80 positive cells as a percentage of total cell number; G: Representative gel blots depicting expression of Tenascin C and Osteopontin‐1; H Tenascin C: and I: Osteopontin‐1. Mean ± SEM, n = 4‐5 mice per group, *P < 0.05 vs FVB group, #*P* < 0.05 vs FVB‐L‐NAME group

To examine if the protective role of metallothionein against L‐NAME‐induced cardiac dysfunction is related to antioxidant capacity, cardiac remodelling, macrophage infiltration and inflammation, levels of antioxidant proteins SOD1 and HMOX‐1, remodelling protein markers tenascin C and osteopontin‐1 and proinflammatory maker IL‐1β were monitored using Western blot analysis, while macrophage infiltration was evaluated by immunohistochemistry. Our results from immunoblot and immunofluorescent staining indicated that L‐NAME led to elevated level of IL‐1β, reduced expression of antioxidant HMOX‐1 and SOD1, without affecting the levels of remodelling protein markers tenascin C and osteopontin‐1. Although metallothionein itself did not elicit any response on these protein markers, it reversed L‐NAME‐induced changes in IL‐1β, SOD1 and HMOX‐1 with little effect on the remodelling protein markers (Figure 8A‐D and G‐I). Interestingly, results of immunofluorescent staining using F4/80 antibody showed that L‐NAME challenge triggered pronounced macrophage infiltration in a comparable manner in both FVB and metallothionein mice (*P* < 0.05 for L‐NAME‐treated groups vs FVB group). Metallothionein itself did not affect macrophage infiltration (Figure 8E‐F).

## 4 DISCUSSION

Salient findings of our present study suggested that the 14‐day L‐NAME treatment elicited experimental hypertension, impaired cardiac contractile function and intracellular Ca^2+ ^handing with little cardiac remodelling, in association with pronounced macrophage infiltration, inflammation, oxidative stress (${\text{O}}_{2}^{-}$ production), loss of mitochondrial membrane potential and increased myocardial apoptosis. In particular, L‐NAME treatment led to cardiac mechanical derangement mainly as manifested by elevated LVESD and LVEDD, decreased fractional shortening, peak shortening, velocity of shortening/relengthening, reduced electrically stimulated fura‐2 fluorescence intensity (ΔFFI) as well as prolonged intracellular Ca^2+^ decay. Furthermore, our study revealed that L‐NAME treatment prompted apoptosis and mitochondrial injury (loss of mitochondrial membrane potential) as manifested by TUNEL staining, Caspase 3 activity and JC‐1 staining. Although L‐NAME challenge itself did not affect the levels of metallothionein in the heart, cardiac overexpression of metallothionein alleviated L‐NAME‐induced cardiac contractile dysfunction with restoration of mitochondrial membrane potential as well as inhibition of inflammation, oxidative stress and apoptosis with little effect on macrophage infiltration. Taken together, these findings suggest a protective role for antioxidants in hypertension‐associated cardiac dysfunction and apoptosis.

Findings from our study suggested a rather important role for apoptosis in L‐NAME ‐induced pathology in the heart. It is well perceived that apoptosis plays a vital role in diverse cardiovascular diseases such as atherosclerosis, obesity, hypertension and various types of cardiomyopathies.^12^, ^39^ Our earlier study has revealed a beneficial role of metallothionein against endoplasmic reticulum (ER) stress‐induced cardiac defects via attenuating myocardial apoptosis.^40^ Results from our current study showed the importance of metallothionein as a potent antioxidant in cardiac dysfunction resulted from L‐NAME‐induced experimental hypertension. Levels of Bax, cleaved Caspase 3, Caspase 9 and Caspase 12 were increased while Bcl‐2 and phosphorylated BAD were suppressed in the heart from L‐NAME‐treated mice. Along with same line, activity assay and TUNEL staining revealed overtly increased Caspase 3 activity and TUNEL‐positive cardiomyocytes, indicating pronounced cell death. Further examination of cardiomyocyte ${\text{O}}_{2}^{-}$ production and mitochondrial membrane potential revealed a significant rise in ${\text{O}}_{2}^{-}$ production along with a drop of ΔΨm following L‐NAME challenge, indicating a role for oxidative stress and mitochondrial injury in L‐NAME‐induced cardiac pathology. In addition, our data further revealed subtle although significant drop in SOD1 and HMOX‐1 following L‐NAME treatment, the effect of which was reconciled by metallothionein, denoting a role for antioxidant defence in L‐NAME‐ and metallothionein‐ induced changes in mitochondrial function. In our hands, the F4/80 macrophage infiltration and elevated IL‐1β levels were both noted following L‐NAME treatment. This is in line with the previous report of overt inflammation in hearts from L‐NAME‐induced hypertensive rats.^41^ Interestingly, the heavy metal scavenger metallothionein only reconciled IL‐1β levels without affecting F4/80 macrophage infiltration. These data likely suggest that inflammation originated from the non‐macrophage sources (such as infiltration of myocardial tissues) 42 perhaps plays a much more predominant role in our L‐NAME‐induced experimental model although further study is warranted. Last but not least, our results showed unchanged levels of cardiac remodelling markers such as tenascin C and osteopontin‐1 under either L‐NAME or metallothionein challenge, implying little role of cardiac remodelling in L‐NAME‐induced hypertension condition (possibly due to a relatively short 2‐week duration of challenge).

Perhaps, the most intriguing data from our work are that the overexpression of the heavy metal antioxidant metallothionein‐attenuated L‐NAME‐induced cardiac contractile dysfunction, superoxide anion production, loss of antioxidant capacity and mitochondrial membrane potential as well as increased apoptosis. Our previous findings demonstrated that metallothionein attenuates lipopolysaccharide‐induced septic cardiomyopathy,^28^ alcoholic cardiomyopathy 43, 44 and diabetic cardiomyopathy.^45^ Furthermore, metallothionein rescued buthionine sulfoximine‐induced oxidative cardiac dysfunction and alleviated cold stress‐induced cardiac anomalies through attenuation of cardiac autophagy.^46^, ^47^ Our finding is consistent with earlier notion that antioxidants can attenuate cardiac dysfunction in hypertension.^9^ Moreover, our recent study revealed that metallothionein can protect heart from L‐NAME‐induced cardiac dysfunction in experimental hypertension through autophagy regulation.^18^ Our data shown here are in favour of the notion that hypertension may impair redox balance and mitochondria function in myocardium, which leads to apoptotic cell death, inflammation and cardiac dysfunction. It is noteworthy that cardiac‐specific overexpression of metallothionein did not affect L‐NAME‐induced rises in both systolic and diastolic blood pressure, suggesting a relatively minor role of coronary endothelial metallothionein for blood pressure regulation although direct effect of vascular metallothionein cannot be ruled out.

### 4.1 Experimental limitations

Although our data suggest that metallothionein rescues against L‐NAME challenge‐induced cardiac functional anomalies, several limitations exist. First and perhaps foremost, expression of metallothionein (type IIa) is non‐physiological and may elicit off‐target effects, making it difficult to evaluate the translational efficacy of this work. Second, the 14‐day L‐NAME‐induced hypertension is somewhat “artificial” and may not recapitulate the genuine pathological changes in hypertensive heart disease derived from chronic essential hypertension. For example, no notable cardiac remodelling was noted in light of the overt presence of macrophages. Thus, special caution should be taken in data interpretation and extrapolation to hypertensive heart diseases.

In summary, our results revealed that the antioxidant metallothionein rescued against cardiac contractile and intracellular Ca^2+^ derangement, inflammation, loss of redox balance (superoxide production and antioxidant defence), mitochondrial injury and apoptosis in a L‐NAME‐induced experimental hypertension model. Given the pivotal role of antioxidants in the regulation of cardiac structure and function, our data favour a role for antioxidants in the management of hypertension‐associated cardiac dysfunction.

## CONFLICT OF INTEREST

The authors declared no conflict of interest for this work.

## AUTHOR CONTRIBUTION

LY, JM and JR designed experiments; LY, JM, YT, QZ and MD performed the experiments; LY, JM and YT analysed the data; LY, WG, LX, JY and JR wrote the manuscript. All authors approved the final manuscript.

## DATA AVAILABILITY

All original data used in this work will be made available upon request.
